# Hepatitis A virus infection: Is it an important hazard to public health? hazards of HAV for public health

**Published:** 2011-04-01

**Authors:** Mehdi Saberifiroozi

**Affiliations:** 1 Digestive Disease Research Center, Tehran University of Medical Sciences, Shariati Hospital, Tehran, IR Iran

**Keywords:** Hepatitis A virus, Seroprevalence, Prevention

Hepatitis A virus (HAV) infection usually spreads through the fecal-oral route. It is an acute, self-limiting disease with a mild course that is often silent in pediatric cases. However, this infection can be more severe and even fatal in older individuals. It usually has an uncomplicated course; however, complications such as cholestatic hepatitis, relapsing hepatitis, extrahepatic manifestations, or rarely, fulminant hepatitis and even death may occur. These complications are more prevalent in adults, especially those more than 40 years of age. Superimposed infection in patients with chronic liver disease, particularly hepatitis B or hepatitis C infection, may accentuate the disease course and cause acute liver failure [1]. The rate of spread and prevalence of infection in each community depends on the hygiene conditions and sanitation status. Because the virus sheds as early as 2 weeks prior to the onset of jaundice, the disease can be transmitted by asymptomatic persons during the incubation period of the virus. Three strategies for HAV prevention have been suggested in high-risk groups. The first strategy is personal hygiene and enteric isolation, including hand washing after close contact. The second strategy is passive immunoprophylaxis via injection of human serum immunoglobulin derived from subjects with high HAV antibody levels. The third route is vaccination, which is usually performed before exposure, but can also be performed in the early post-exposure period in outbreaks [2].

Active immunoprophylaxis by vaccination is the most important strategy for preventing disease at the national level. To implement a national HAV vaccination campaign, the cost and an epidemiological survey for this infection should be considered [3]. Vaccination is very effective for preventing this disease; however, due to limited health budgets, evaluation of the cost-effectiveness in each region is very important. In Middle East countries with high or intermediate HAV infection prevalence, multiple strategies can be implemented to prevent HAV infection. The success of routine vaccination, accelerated vaccinations in outbreaks, ongoing vaccination of children, and outbreak-control vaccination programs largely depends on the feasibility of rapidly vaccinating the target population [2]. Proper decision-making regarding the need for national vaccination depends on the prevalence and incidence of HAV infection. In this issue of Hepatitis Monthly, Taghavi et al. have reported the prevalence of HAV infection in unmarried young adults in the Fars province of southern Iran [4].

In this study, 88.2% of 1050 subjects were seropositive for the anti-HAV antibody, whereas 11.8% were negative for it. Anti-HAV antibody positivity was observed in 79.3% of subjects < 20 years old and 99% of those > 30 years old (p = 0.01). Anti-HAV positivity was significantly more frequent in individuals living in rural regions (95.9% of individuals in rural regions vs. 85.1% of individuals living in urban areas). Family crowding was associated with higher antibody levels. Previously, Iran was considered to have a high rate of HAV infection. However, because the hygiene conditions, sanitation status, and education levels have improved dramatically in the last 2 decades, lower rates of infection have been observed [5]. Despite high rate of HAV infection in most studies from different counties, in some studies very low rate of exposure was reported, which may be due to inclusion of children and adolescent and young adult groups.

The rate of seropositivity for anti-HAV IgG has varied from 8.6% among 816 subjects between the age of 6 and >50 years [6], up to 22.3% among 1018 children aged 6 months to 14.9 years in Tehran [7], 61.6% among 1065 subjects aged 6 months to 20 years [8], 44.3% among 300 children in Zanjan [9], 50% in Tehran [10], 68% in Shiraz [11], 86.8% in Golestan [12], 89.5% in Yazd [13], 84.9% in Sari [14], 98.6% in Golestan [15], 94.9% in Qazvin [16], 86% in Tehran [17], and 97.63% in soldiers in Tehran [18]. Merat and colleagues performed a serological assay to determine the prevalence of HAV infection among 1896 subjects aged 18 to 65 years in Tehran, Golestan in the north, and Hormozgan in the south, and overall, 86% of the subjects were positive for anti-HAV IgG. The rate of infection in younger subjects in urban areas was 70% [19].

Patients with chronic liver disease may experience exacerbation of liver disease and even a fatal clinical course if a superimposed HAV infection occurs. The rate of HAV immunity in patients with chronic liver disease has been determined in a few studies. In 3 studies, the rates of anti-HAV seropositivity were greater than 95% in patients aged > 30 years, 71.4% in patients aged 10-20 years, and 59.4% in patients aged 10-20 years [20][21][22]. Mhoghani et al. reported a 80% rate of HAV immunity in chronic hepatitis B patients with different stages of disease. The mean age of patients with anti-HAV positivity (40.91 ± 12.91 years) was significantly higher than that of patients with negative antibody status (33.49 ± 12.88 years, p = 0.000). They suggested vaccination in patients with chronic liver disease [23].

The improvement of sanitation and national health programs in some countries in the Middle East and Asia reduced the rate of HAV infection in children, and as a result, more young adults are prone to this infection. This has been observed in many communities as the epidemiology of HAV shifted from a hyperendemic to a hypoendemic pattern. In a report from India, in 1932 patients with acute viral hepatitis, 11.4% of the cases were related to HAV. Over a 5-year period, the rate of infection in children increased from 10.6% to 22.0% and the rate of infection in adultsincreased from 3.4% to 12.3% [24]. According to a report from South Korea, the rate of anti-HAV antibody positivity has decreased, and the rate of acute HAV infection has increased in subjects aged 10-50 years during the past 3 decades [25]. In a multi-center study from Lebanon, HAV seroprevalence was 78% in subjects = 21 years, 28% in subjects aged 6-10 years, and 11% in subjects aged 1-5 years. Due to the low rate of HAV immunity and more severe form of HAV infection in adults and children, the authors concluded that a national vaccination campaign was required [26]. Some reports have indicated an increasing rate of acute HAV infection in adults in Iran in recent years [27][28][29]. Reports in our country and others in Asia implied that HAV infection will be an important health problem in the future. Health status differences among neighbors traveling between these countries may result in the transmission of hepatitis to susceptible subjects [30]. As a whole, the findings imply that in the near future, more cases of acute HAV infection in children and adults will be reported. HAV infection can be effectively prevented by HAV vaccines [31]. Generally, susceptibility to HAV infection is higher in people younger than 20 years old both for healthy subjects and for patients with chronic liver disease. Considering the increased susceptibility of young and adults to acute HAV infection in Asian countries, some suggestions can be proposed. First, the available data should be discussed by a team of experts. If needed, well-designed surveys should be performed to estimate the exact prevalence and incidence of this viral illness in our community. Second, according to the present data and/or future research, the possible benefits of national childhood vaccination programs for viruses such as hepatitis B or a catch-up vaccination program for the high school age group should be considered. This strategy can effectively decrease the incidence of HAV infection. Some authors have suggested a possible benefit only in young subjects in selected urban regions and have not recommended routine vaccination of all people [19]. Third, the health system infrastructure should be prepared to handle possible outbreaks at daycare centers or nurseries or common source outbreaks such as those caused by contaminated foods or water. This event requires the early diagnosis of infected subjects and accelerated vaccination of exposed or susceptible persons. Proper vaccination of high-risk groups such as patients with chronic liver disease, especially those younger than 20-30 years old, is suggested. The Ministry of Health is urged to report HAV infection (clinical) rates to improve the interpretation of the HAV situation in our country.
